# The Theory and Practice of the Ophthalmoscope

**Published:** 1888-10

**Authors:** 


**The Theory and Practice of the Ophthalmoscope.** By JOHN HERBERT CLAIBORNE, M. D. Same.

These little brochures are two numbers of Series III. of the Physician's Leisure Library. They are as creditable productions, in a scientific sense, as could be well condensed into such small space, while the press-work is unexcelled by any cheap editions of medical literature that we have seen. The publisher is to be commended for his enterprise in placing such choice material within the reach of the most limited purse, and the authors for their skill in conveying so much information that is useful, within such narrow limits.

